# Cough quality in children: a comparison of subjective vs. bronchoscopic findings

**DOI:** 10.1186/1465-9921-6-3

**Published:** 2005-01-08

**Authors:** Anne Bernadette Chang, Justin Thomas Gaffney, Matthew Michael Eastburn, Joan Faoagali, Nancy C Cox, Ian Brent Masters

**Affiliations:** 1 Dept of Paediatrics & Child Health, University of Queensland; Dept Respiratory Medicine, Royal Children's Hospital, Brisbane, Qld 4029, Australia; 2 Department of Respiratory Medicine, Royal Children's Hospital,, Herston Rd, Brisbane, Qld 4029, Australia; 3 School of Information Technology and Electrical Engineering, University of Queensland, St Lucia, Qld, Australia; 4 Department of Microbiology, Queensland Health Pathology Service, Royal Brisbane Hospital, Herston, Qld 4029, Australia; 5 Department of Cytology, Queensland Health Pathology Service, Royal Brisbane Hospital, Herston, Qld 4029, Australia; 6 Dept Respiratory Medicine, Royal Children's Hospital, Herston Rd, Brisbane, Qld 4029, Australia

## Abstract

**Background:**

Cough is the most common symptom presenting to doctors. The quality of cough (productive or wet vs dry) is used clinically as well as in epidemiology and clinical research. There is however no data on the validity of cough quality descriptors. The study aims were to compare (1) cough quality (wet/dry and brassy/non-brassy) to bronchoscopic findings of secretions and tracheomalacia respectively and, (2) parent's vs clinician's evaluation of the cough quality (wet/dry).

**Methods:**

Cough quality of children (without a known underlying respiratory disease) undergoing elective bronchoscopy was independently evaluated by clinicians and parents. A 'blinded' clinician scored the secretions seen at bronchoscopy on pre-determined criteria and graded (1 to 6). Kappa (K) statistics was used for agreement, and inter-rater and intra-rater agreement examined on digitally recorded cough. A receiver operating characteristic (ROC) curve was used to determine if cough quality related to amount of airway secretions present at bronchoscopy.

**Results:**

Median age of the 106 children (62 boys, 44 girls) enrolled was 2.6 years (IQR 5.7). Parent's assessment of cough quality (wet/dry) agreed with clinicians' (K = 0.75, 95%CI 0.58–0.93). When compared to bronchoscopy (bronchoscopic secretion grade 4), clinicians' cough assessment had the highest sensitivity (0.75) and specificity (0.79) and were marginally better than parent(s). The area under the ROC curve was 0.85 (95%CI 0.77–0.92). Intra-observer (K = 1.0) and inter-clinician agreement for wet/dry cough (K = 0.88, 95%CI 0.82–0.94) was very good. Weighted K for inter-rater agreement for bronchoscopic secretion grades was 0.95 (95%CI 0.87–1). Sensitivity and specificity for brassy cough (for tracheomalacia) were 0.57 and 0.81 respectively. K for both intra and inter-observer clinician agreement for brassy cough was 0.79 (95%CI 0.73–0.86).

**Conclusions:**

Dry and wet cough in children, as determined by clinicians and parents has good clinical validity. Clinicians should however be cognisant that children with dry cough may have minimal to mild airway secretions. Brassy cough determined by respiratory physicians is highly specific for tracheomalacia.

## Background

Cough is the most common symptom presenting to medical practitioners in Australia, the UK and USA [1-3]. Cough quality, specifically dry versus we t[4] or productive cough, is often used in epidemiological [5-7] and clinical research [8,9]. Clinically, physicians also often differentiate between dry and wet cough [10-12] but there are no studies that have evaluated if these are reproducible descriptors. In adults, productive cough is usually obvious but children however often swallow their sputum and hence a 'wet cough' is used inter-changeably with 'productive cough' to describe cough quality in young children who are unable to expectorate [10,13]. It is known that nocturnal cough is unreliably reported in both children [14] and adults [15] but there is no data on cough quality. Wet and dry cough are determined subjectively as there are no 'gold standards'. To date there are no human studies that have identified the objective relationship of the cough descriptors to mucus secretory states.

The sound of a cough is due to vibration of larger airways and laryngeal structures during turbulent flow in expiration [16,17]. It is not known which generation of the airways is involved when the human ear identifies a wet cough and currently there are no validated human models that allow measurement of increased airway mucus. Mucus hypersecretory states in human diseases can occur from a variety of mechanisms which include; hypersecretion of stored mucin, hypertrophy or hyperplasia of goblet cells and/or increased synthesis from over-expression of mucin genes [18]. In disease states, it is not known which mechanism or site of production is the most important but in smokers with chronic bronchitis, a common cause of productive cough in adults, the larger bronchi (bronchi of diameter >4 mm ie segmental bronchi and above) [19] are the site of greatest inflammation [18]. Flexible bronchoscopy allows an *in-vivo *visual assessment of larger airways usually to the 3^rd ^(lobar bronchi) or 4^th ^generation (segmental bronchi) in young children.

The study aims were to compare (1) cough quality (wet vs dry and brassy vs non-brassy) with bronchoscopic findings of secretions and tracheomalacia respectively and, (2) parent(s) vs clinician's evaluation of the cough quality (wet and dry). We hypothesised that clinical assessment of cough is good compared to bronchoscopic findings and that a wet cough is related to presence of airway secretions.

## Methods

Children electively admitted for bronchoscopy without a known underlying respiratory diagnosis were seen by a member of the research team 0.5–3 hours prior to bronchoscopy. The clinician's assessment of cough quality (wet or dry) was recorded on a standardised sheet (based on the cough present on the day of the bronchoscopy), before the parent(s) independently evaluated the current (the morning of, or last 12 hours) cough quality (wet or dry) of their child. For clinician's assessment of wet/dry cough, when no spontaneous cough was heard or if child was too young to elicit a cough, cough quality (wet or dry) was deemed 'non-assessable'. Clinicians also rated cough as 'brassy' or 'non-brassy' based on coughs heard anytime before bronchoscopy. For assessment of reliability of cough quality (wet/dry and brassy/non-brassy), 21 cooperative children had their coughs digitally recorded (Acer Pocket PC n11, Taiwan) using music compact disc quality format (44.1 kHz, 16 bit) on the morning of their bronchoscopy. These stored cough sounds were later replayed (using headphones 30–10,000 Hz, Lanier, Japan) from a computer and re-scored in a blinded manner (blinded to the child's name and cough quality assigned earlier) for wet/dry and brassy/non-brassy qualities. Written consent was obtained from a parent and the study approved by the hospital's ethics committee on human research.

### Bronchoscopy and quantification of secretions seen during bronchoscopy

Flexible bronchoscopy was performed under general anaesthesia as previously described [20-22]. Briefly, anaesthesia was induced with sevoflurane in 100% oxygen administered through a Jackson Rees T piece circuit, the vocal cords and upper trachea then sprayed (4 mg/kg lignocaine via a Cass needle). Atropine was given intravenously to most children aged <12 months. In all children a video flexible bronchoscope (BF 3C160, Olympus, Tokyo, Japan) entered the circuit via the port of a swivel right angle connector attached to a facemask. Images were projected onto a monitor (Sony Trinitron, Tokyo, Japan).

**Figure 1:**
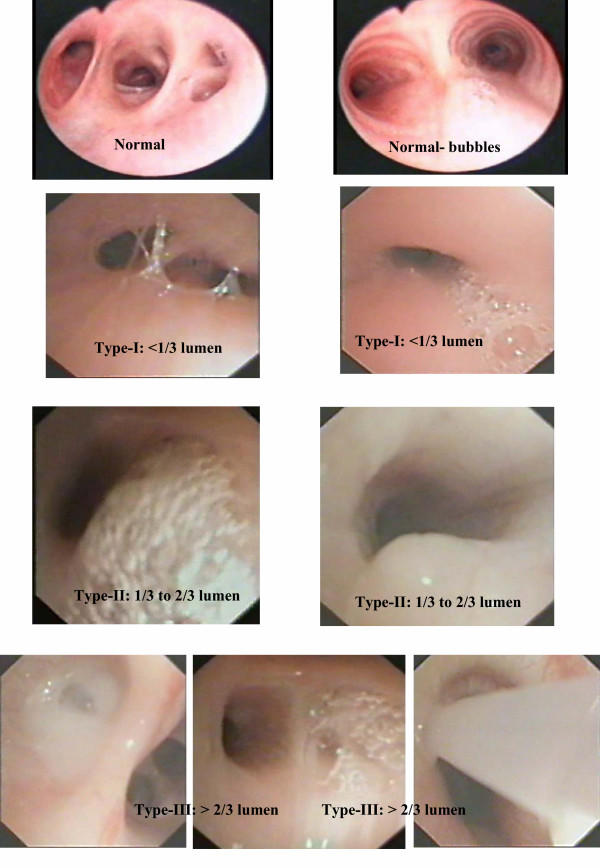
Bronchoscopic secretion quantification card.

A respiratory consultant (ABC or IBM) blinded to the child's history and cough quality scored the bronchoscopy sheet quantifying the amount of secretions at the time of the bronchoscopy in real time. When no scorer was available, the session was videotaped and played back. A secretion quantification card (figure 1) was visible to the scorer at all times. Secretions were quantified according to amount of mucus in the airways in relation to lumen size (fig 1) and scored from the trachea to the level of lobar bronchi (total of 9; trachea, right main stem, right upper lobe, right middle lobe, right lower lobe, left main stem, left upper lobe, left lingula, left lower lobe). When segmental bronchi were seen, the worst segment (ie segment with most secretions) was scored. These scores were used to obtain a final grade of bronchoscopic secretions (BS) from grades 1 to 6;

BS Grade 1 = Nil secretions

BS Grade 2 = Near dry = Bubbles only in < half total number of bronchi involved

BS Grade 3 = Minimal = Bubbles found in > half total number of bronchi involved or Secretion type-I in < half total number of bronchi involved

BS Grade 4 = Mild = Secretion type-I, > half total number of bronchi involved or Secretion type-II, < half total number of bronchi involved

BS Grade 5 = Mod = Secretion type-II, > half total number of bronchi involved or Secretion type-III, < half total number of bronchi involved

BS Grade 6 = Large = Secretion type-III, > half total number of bronchi involved Inter-rater reliability of BS grading was assessed by replaying the videotapes of the recorded bronchoscopy of 20 children, with the 2^nd ^assessor blinded to the child's condition.

BAL was obtained from the macroscopically most abnormal lobe; when changes were generalised, BAL was obtained from the right middle lobe. Cell count was performed on the cell suspension, cytocentrifuge slides were prepared and stained (modified Wright's stain) for cell differential profile. All cellular examinations were performed by cytologists blinded to the children's medical history.

### Statistics

Data were not normally distributed and thus non parametric analyses were used; medians and inter-quartile range (IQR) were used for all descriptive data and Kruskal Wallis for comparisons between groups. Cohen's kappa (K) with 95%CI was utilised for inter and intra-observer reliability and graded from 'poor' (K<0.2) to 'very good' (K = 0.81–1.0)[23]. For calculation of sensitivity and specificity, negative and positive predictive values (NPV, PPV); cough quality was assigned to dry when a history of cough was absent and bronchoscopy findings at two cut offs (grades 3 and 4) of BS grades were taken as the 'gold standard' eg for cut-off at BS grade 3, BS grades 1–2 were defined as no secretions and BS grades = 3 defined as secretions present. To determine if cough quality (wet/dry) was predictive of amount of secretions found during bronchoscopy, a receiver operating characteristic (ROC) curve was generated [24] where cough quality wet/dry was considered the true positive/negative and the bronchoscopic secretion scoring (1 to 6) as the ordinal rating scale. Two tailed p value of < 0.05 was considered significant. SPSS ver 11.1 was utilised for most statistical calculation.

## Results

Median age of the 106 children (62 boys, 44 girls) enrolled was 2.6 years (IQR 5.7). Indications for bronchoscopy were chronic cough (n = 44, 41.5%), wheeze (n = 21, 19.8%), stridor (n = 16, 15.4%), investigation of persistent radiological changes (n = 14, 13.5%), recurrent pneumonia (n = 6, 5.8%), suspicion of aspiration lung disease (n = 3, 2.9%), BAL and suspected foreign body (n = 1 each, 2%). In four children, BS grades were not obtained (session was inadvertently not recorded and 'blinded' clinician not present at bronchoscopy). Scores of BS were done in real time in all but 9 children.

**Figure 2:**
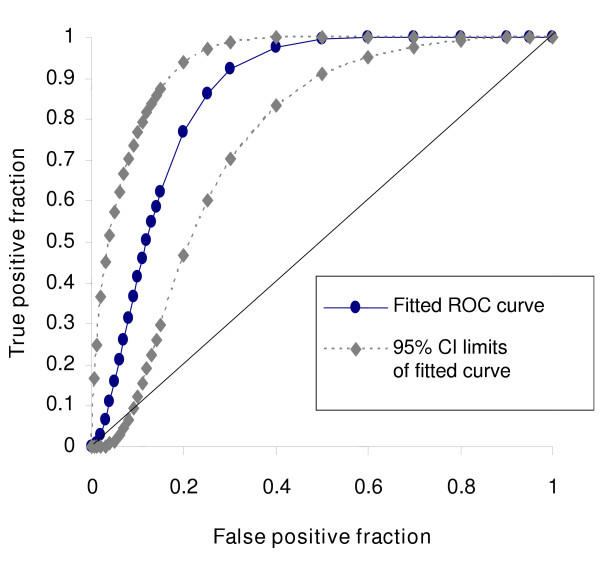
ROC curve with 95%CI relating cough quality (wet/dry) to bronchoscopic secretion (BS) grades from 1–6.

In 30 children, cough was non-assessable. Agreement between clinicians and parents assessment of cough quality (wet/dry) was good (K = 0.75, 95%CI 0.58, 0.93). For cough quality of 'wet/dry', cough assessed by clinicians had the highest specificity, sensitivity, NPV, PPV and positive likelihood ratio for both BS cut-offs (tables 1 and 2). Parent(s) assessment were less precise but only marginally so. The area under the fitted ROC curve (figure 2) was 0.85 95%CI 0.77, 0.92. The specificity, NPV and likelihood ratio for brassy cough assessed against gold standard bronchoscopic finding of tracheomalacia was good (table 1) but less than that for cough quality of wet/dry.

**Table 1:** Assessment of cough quality vs bronchoscopic findings with BS cut off at grade 3*

**Assessment type (clinical vs bronchoscopic findings)**	**Sensitivity**	**Specificity**	**NPV**	**PPV**	**Positive LR**
**Clinician**	1.00	0.55	1	0.64	2.21
Cough quality (wet/dry)					
assessed by clinician (n = 96)					
**Parent(s)**	0.95	0.54	0.93	0.61	2.06
Cough quality (wet/dry)					
assessed by parents (n = 92)					
Combined*(n = 100)	0.98	0.54	0.97	0.62	2.10
**Tracheomalacia **(n = 81)#	0.57	0.81	0.84	0.52	3.12

*Cough quality (wet/dry) assessed by clinicians combined with parents. When cough was non-assessable by clinician and child has current cough, parental assessment of the cough (wet or dry) was taken. If child has no history of current cough, cough was assigned 'dry'.

LR = likelihood ratio.

Specificity, sensitivity of dry and wet cough was assessed against bronchoscopic findings as the gold standard where *BS grades ≥ 3 were considered abnormal (secretions present) and ≤ 2 considered normal (no secretions). #That for tracheomalacia was assessed using clinicians record of presence/absence of brassy cough with bronchoscopic findings of tracheomalacia.[21]

**Table 2:** Assessment of cough quality vs bronchoscopic findings with BS cut off at grade 4*

**Assessment type (clinical vs bronchoscopic findings)**	**Sensitivity**	**Specificity**	**NPV**	**PPV**	**Positive LR**
**Clinician**	0.79	0.75	0.82	0.72	3.22
Cough quality (wet or dry)					
assessed by clinician (n = 96)					
**Parent(s)**	0.78	0.71	0.80	0.67	2.69
Cough quality (wet or dry)					
assessed by parents (n = 92)					
Combined* (n = 100)	0.77	0.73	0.80	0.69	2.88

*Cough quality (wet/dry) assessed by clinicians combined with parents. When cough was non-assessable by clinician and child has current cough, parental assessment of the cough (wet or dry) was taken. If child has no history of current cough, cough was assigned 'dry'.

LR = likelihood ratio.

Specificity, sensitivity of dry and wet cough was assessed against bronchoscopic findings as the gold standard where BS grades ≥ 4 were considered abnormal (secretions present) and ≤ 3 considered normal (no secretions).

There was little difference in sensitivity and specificity between children grouped by indication for bronchoscopy (cough or other indications). Values were marginally better in older children (tables 4 and 5 in supplementary data additional file 2). Area under the fitted ROC curve was similar for both age groups (aged ≤ 2 years = 0.811, 95%CI 0.79, 0.84; age >2 = 0.84, 95%CI 0.74, 0.95). Agreement for clinicians vs parents cough quality (dry/wet) was better in children aged ≤ 2 years (K = 0.85, 95%CI 0.57, 1.0; n = 42 but 18 non-assessable) than that for those age >2 years (K = 0.70, 95%CI 0.49, 0.92; n = 64, but 12 non-assessable) (see additional file 1).

Using recorded coughs, kappa scores were 'very good' for both intra-observer and inter-clinician agreement for wet and dry cough (K = 1.0 and 0.88 [95%CI 0.82–0.94] respectively). There was only one disagreement in wet and dry cough between clinicians and in this child the cough was mildly wet (BS grade of 3). Kappa scores for intra-observer and inter-observer clinician agreement for brassy cough was good, K in both was 0.79, 95%CI 0.73, 0.86. Inter-rater agreement for BS grades was 'very good' (weighted K = 0.95, 95%CI 0.87–1).

Cellularity for total cell count, percentages of neutrophils and macrophages were significantly different between children grouped by BS grade cut-offs of 3 and 4 as well as wet/dry cough (table 3).

**Table 3:** Cellular differential profile in BALs

**Median**	**TCC (IQR)**	**% M IQR)**	**% N (IQR)**	**% Lym (IQR)**	**% Eos (IQR)**
**BS cutoff at grade 3**					
≤2 (n = 31)	195 (290)	82.0 (15.8)	5.0 (7)	13.5 (15.8)	0 (0)
≥3 (n = 70)	334.0 (425)	66.0 (45)	12.0 (38)	11.0 (16.0)	0 (0)
p value^	0.038	0.001	0.006	0.605	0.758
					
**BS cutoff at grade 4**					
≤3 (n = 52)	176 (257)	81.0 (17.0)	6.0 (8.0)	13.0 (16.0)	0 (0)
≥4 (n = 49)	368 (574)	51.5 (59.8)	20.0 (47.0)	11.0 (15.0)	0 (5)
p value^	0.0001	0.0001	0.0001	0.445	0.613
					
Cough quality*					
Wet (n = 45)	365 (522)	51.5 (49.8)	25.0 (43)	13.0 (16)	0 0
Dry (n = 25)	176 (315)	80.5 (24.8))	5.5 (13.0)	1.8 (16.0)	0 (0)
No history (n = 28)	80 (310)	15 (16.5)	1 (7.5)	1 (11.5)	0 (0)
	310	16.5	7.5	11.5	0
p value^	0.017	0.0001	0.001	0.242	0.769

^p value = examined using Kruskal Wallis test.

*assessed by clinician

TCC = total cell count; N = neutrophils, M = macrophages, L = lymphocytes, Eos = eosinophils,

## Discussion

We have shown that clinical assessment of cough quality of wet/dry cough generally relates to bronchoscopic secretions determined using a standardised scoring system (BS grades). When cough is wet, secretions were always present; when cough was dry secretions if present, were usually minimal or mild. Clinicians were marginally better than parents at assessing wet/dry cough and agreement between the 2 groups was good. When clinicians detected presence of a brassy cough, tracheomalacia was usually present. Inter-rater clinician agreement for cough qualities of dry/wet and brassy/non brassy was good.

Accuracy and reliability of symptoms are important in clinical and research settings. Cane and colleagues [25,26]. found that parental reports of wheeze and stridor are often not accurately reported in a clinic setting. There is no data on the validity of cough quality in spite its use in management and diagnostic guidelines [11,27,28] and cough being the most common symptom seen by general practitioners [1-3]. The level of agreement recommended for symptoms and signs to be used in clinical prediction rules is kappa value of ≥ 0.6 [29]. The kappa values we obtained in this study well exceeded 0.6. Specifically, intra and inter-clinician evaluation was very good and parental reporting of cough quality (wet/dry) also related well to clinicians' evaluation.

When compared to bronchoscopic findings, this study showed that a wet cough is always associated with BS grades of 3 or more. Dry cough is less valid; the presence of dry cough does not necessary indicate absence of secretions. However BS grades are less in dry cough as shown in the ROC curve. The generation of cough sounds and some factors that influence cough sounds have been examined in the laboratory [16,30]. Using cough sound analysis (spectrogram and time-expanded waveform), productive and non-productive cough can be differentiated in the laboratory [30]. However to date there is no data on its clinical reliability and its relationship to quantification of airway secretions. In humans, it is not known how much mucus is required and where it has to be located for the human ear to detect presence of a moist cough. It is likely that mucus in the large airways is required for detectable difference in cough quality as the sound of cough is generated from vibration of larger airways and laryngeal structures during turbulent flow in expiration [16,17]. Laminar airflow, which occurs in smaller airways, is inaudible [31]. In an animal model, Korpas and colleagues showed that a certain amount of mucus is required to alter cough sound; 0.5 ml of mucus instilled into the trachea of cats altered cough sound, too little mucin had no effect on cough quality whilst too much mucin impaired breathing [32]. Our study findings support this and it is not surprising that when the cough is dry, BS grades were less. The rheological properties of airway mucus also influence cough sound [17]. It is not known how airway secretions in the more peripheral airways influences the sound of cough.

One possible limiting factor of our study is the choice of cut offs for BS grades in determining presence or absence of significant secretions. We chose to use a cut off of 3 as a minor amount of bubbles in the airways can be present from trickling of lignocaine into the airways or spillage from the upper airways. BS cut-off at grade 4 resulted in improved specificity but decreased sensitivity. Children grouped by both BS cut-offs (3 and 4) had significantly different airway cellular profile. The clinical significance of minimal BS grades and appropriate cut-offs can only be determined in a prospective follow-up study which is not an aim of this study. This study did determine that our BS scoring method was easy to use (most done in real time) and had very good inter-rater agreement. The clinical outcomes of wet and dry cough were not the aims of this study and thus cannot be determined here. To relate clinical outcomes to cough descriptors would ideally require a randomised controlled trial with dry and wet cough as entry criteria. A follow-up cohort study with strict clinical diagnostic categories would be useful and we have shown in a preliminary study that dry cough was significantly more likely to naturally resolve than wet cough [33].

In addition to the limitation of quantifying airway secretions using a bronchoscopic method, this study is also limited by a number of factors. Firstly, clinical repeatability or agreement of cough sounds was assessed by doctors in a tertiary setting. Whether or not these findings can be extrapolated to the secondary and primary setting can only be speculated. Hay and colleagues showed that inter-observer agreement for clinical signs of fever, tachypnoea and chest signs were poor to fair (kappa of 0.12–0.39) in the primary care setting but these signs are known to have good agreement in secondary care settings [34]. However as parents were almost as good as clinicians in our study and are 'untrained' compared to medical practitioners, we would expect that this data can be extrapolated to most primary and secondary settings. Secondly, anaesthesia and atropine could possibly influence mucus quantity and properties. However this influence, if any, is likely to be small as both bronchoscopists (ABC, IBM) are experienced (our recorded average total theatre time is relatively short at 22 mins) [22], and atropine is given just immediately prior to commencement of bronchoscopy.

Determining the validity of cough quality in children is important not only because of the commonality of the clinical problem of cough but also its use in guidelines and research studies [11,27,28]. A particularly important finding is the presence of small amounts of secretions in children with dry cough which may have implications in the management of suppurative lung disease; a dry cough may represent early disease process where only a small amount of mucous is present.

## Conclusion

We conclude that the description of a cough as wet or dry cough as determined by clinicians and parents has good clinical validity as it has good agreement with, and relates to, quantification of airway secretions. However as minimal amount of secretions may be present in children with dry cough, clinicians should be cognisant that a dry cough may eventually become wet if airway secretions increase. Thus it should not be assumed that airway secretions are absent in children with chronic dry cough and cough quality in these children should be reviewed. We also conclude that the brassy cough determined by respiratory physicians is highly specific for presence of tracheomalacia.

## List of Abbreviations

BAL Bronchoalveolar lavage

BS Bronchoscopic secretion

K Kappa

NPV Negative predictive value

PPV Positive predictive value

ROC receiver operating characteristic

## Authors' contributions

AC conceived the idea, designed the study, performed the data analysis and drafted the manuscript. JG participated in data acquisition and coordination of project. ME participated in electronic acquisition of data and software for sound recordings. JF and NC designed the microbiology and cytological components respectively and both helped draft the manuscript. IBM helped in formulation of overall study design, data acquisition and drafting of the manuscript. All authors read and approved the manuscript.

## Supplementary Material

Additional File 1 **Figure 3: ROC curve **ROC curve with 95%CI relating cough quality (wet/dry) to bronchoscopic secretion (BS) grades from 1–6 in children grouped according into age (a) ≤ 2 years and (b) > 2 years. Click here for file

Additional File 2 **Table 4: Assessment of cough quality vs bronchoscopic findings in children grouped by indication for bronchoscopy **4a: Assessment of cough quality vs bronchoscopic findings in children whose indication for bronchoscopy was cough 4b: Assessment of cough quality vs bronchoscopic findings in children whose indication for bronchoscopy was others (ie not cough) **Table 5: Assessment of cough quality vs bronchoscopic findings in children grouped by age **5a: Assessment of cough quality vs bronchoscopic findings in children aged ≤ 2 years 5b: Assessment of cough quality vs bronchoscopic findings in children aged > 2 years Click here for file
