# Supplementary material for: Cough quality in children: a comparison of subjective vs. bronchoscopic findings
Source: Respir Res. 2005 Jan 8;6(1):3. doi: 10.1186/1465-9921-6-3 (PMC545936; doi:10.1186/1465-9921-6-3)
Supplement: Additional File 1 — Figure 3: ROC curve ROC curve with 95%CI relating cough quality (wet/dry) to bronchoscopic secretion (BS) grades from 1–6 in children grouped according into age (a) ≤ 2 years and (b) > 2 years. [file 1465-9921-6-3-S1.ppt]

## Slide 1
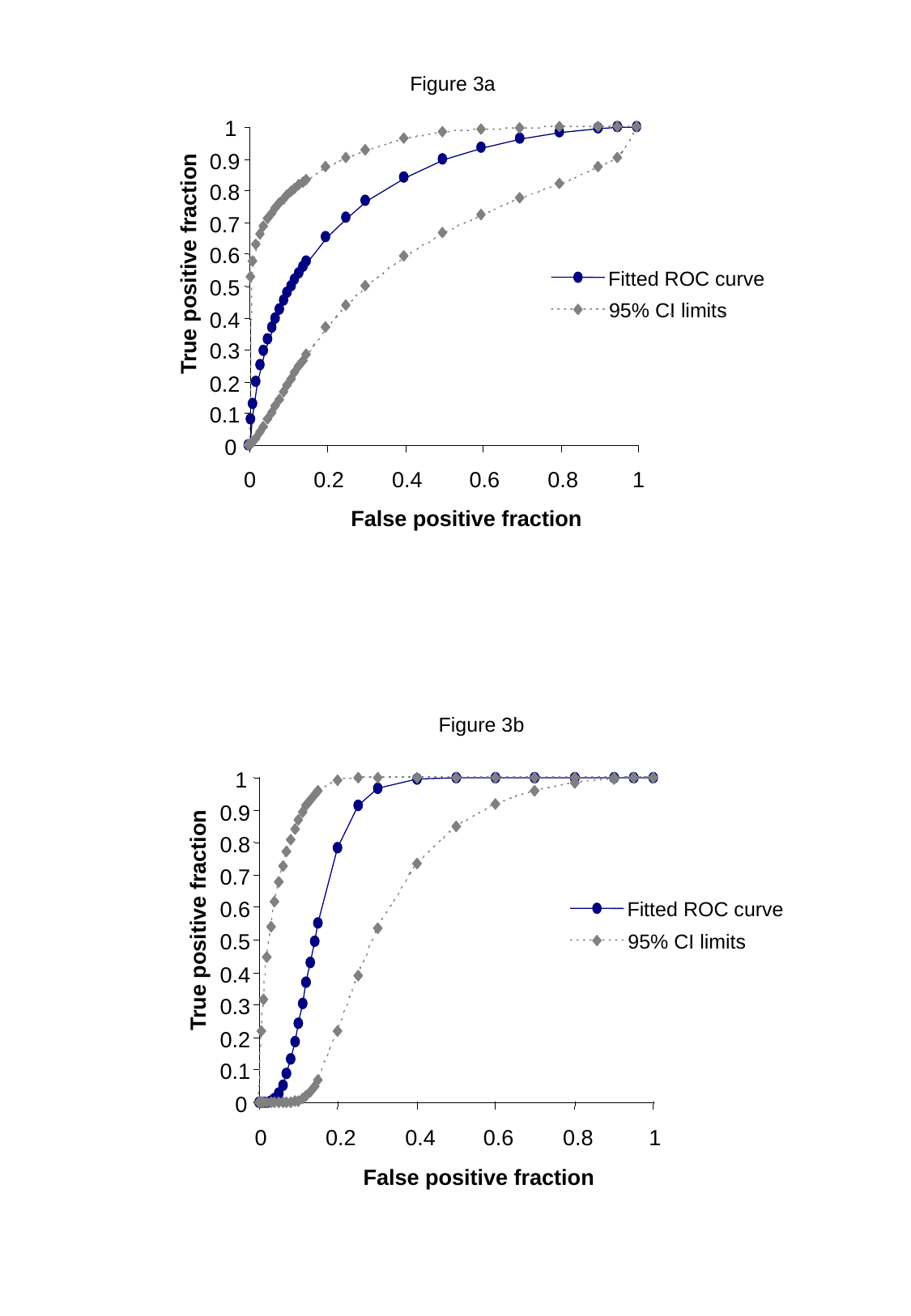

Figure 3a
1
0.9
0.8
0.7
0.6
True positive fraction
Fitted ROC curve
95% CI limits
0.5
0.4
0.3
0.2
0.1
0
0
0.2
0.4
0.6
0.8
1
False positive fraction
Figure 3b
1
0.9
0.8
0.7
0.6
Fitted ROC curve
95% CI limits
True positive fraction
0.5
0.4
0.3
0.2
0.1
0
0
0.2
0.4
0.6
0.8
1
False positive fraction
