# Supplementary material for: Cough quality in children: a comparison of subjective vs. bronchoscopic findings
Source: Respir Res. 2005 Jan 8;6(1):3. doi: 10.1186/1465-9921-6-3 (PMC545936; doi:10.1186/1465-9921-6-3)
Supplement: Additional File 2 — Table 4: Assessment of cough quality vs bronchoscopic findings in children grouped by indication for bronchoscopy 4a: Assessment of cough quality vs bronchoscopic findings in children whose indication for bronchoscopy was cough 4b: Assessment of cough quality vs bronchoscopic findings in children whose indication for bronchoscopy was others (ie not cough) Table 5: Assessment of cough quality vs bronchoscopic findings in children grouped by age 5a: Assessment of cough quality vs bronchoscopic findings in children aged ≤ 2 years 5b: Assessment of cough quality vs bronchoscopic findings in children aged > 2 years [file 1465-9921-6-3-S2.doc]

**Table 4a: Assessment of cough quality vs bronchoscopic findings in children whose indication for bronchoscopy was cough**

| **Assessment type**  **(clinical vs bronchoscopic findings)** | **Sensitivity** | **Specificity** | **NPV** | **PPV** | **Positive LR** |
| --- | --- | --- | --- | --- | --- |
| **Clinician**  Cough quality (wet/dry) assessed by clinician (n=40) | 1 | 0.57 | 1 | 0.81 | 2.33 |
| **Parent(s)**  Cough quality (wet/dry) assessed by parents (n=36) | 0.96 | 0.3 | 0.75 | 0.78 | 1.37 |
| **Combined** (n=41) | 0.96 | 0.57 | 0.81 | 0.89 | 2.25 |
| Cough quality (wet/dry) assessed by clinicians combined with parents. When cough was non-assessable by clinician and child has current cough, parental assessment of the cough (wet or dry) was taken. If child has no history of current cough, cough was assigned ‘dry’ | | | | | |

LR=likelihood ratio.

Specificity, sensitivity of dry and wet cough was assessed against bronchoscopic findings as the gold standard where BS grades ≥3 were considered abnormal (secretions present) and ≤2 considered normal (no secretions).

**Table 4b: Assessment of cough quality vs bronchoscopic findings in children whose indication for bronchoscopy was others (ie not cough)**

| **Assessment type**  **(clinical vs bronchoscopic findings)** | **Sensitivity** | **Specificity** | **NPV** | **PPV** | **Positive LR** |
| --- | --- | --- | --- | --- | --- |
| **Clinician**  Cough quality (wet/dry) assessed by clinician (n=56) | 1 | 0.54 | 1 | 0.49 | 2.12 |
| **Parent(s)**  Cough quality (wet/dry) assessed by parents (n=56) | 0.93 | 0.5 | 0.95 | 0.38 | 1.86 |
| **Combined** (n=59) | 1 | 0.51 | 1 | 0.45 | 2.05 |
| Cough quality (wet/dry) assessed by clinicians combined with parents. When cough was non-assessable by clinician and child has current cough, parental assessment of the cough (wet or dry) was taken. If child has no history of current cough, cough was assigned ‘dry’ | | | | | |

LR=likelihood ratio.

Specificity, sensitivity of dry and wet cough was assessed against bronchoscopic findings as the gold standard where BS grades ≥3 were considered abnormal (secretions present) and ≤2 considered normal (no secretions).

**Table 5a:**

**Assessment of cough quality vs bronchoscopic findings in children aged ≤ 2 years**

| **Assessment type**  **(clinical vs bronchoscopic findings)** | **Sensitivity** | **Specificity** | **NPV** | **PPV** | **Positive LR** |
| --- | --- | --- | --- | --- | --- |
| **Clinician**  Cough quality (wet/dry) assessed by clinician (n=34) | 1 | 0.42 | 1 | 0.57 | 1.73 |
| **Parent(s)**  Cough quality (wet/dry) assessed by parents (n=34) | 0.94 | 0.38 | 0.89 | 0.48 | 1.49 |
| **Combined** (n=39) | 0.94 | 0.41 | 0.9 | 0.55 | 1.59 |
| Cough quality (wet/dry) assessed by clinicians combined with parents. When cough was non-assessable by clinician and child has current cough, parental assessment of the cough (wet or dry) was taken. If child has no history of current cough, cough was assigned ‘dry’ | | | | | |

LR=likelihood ratio.

Specificity, sensitivity of dry and wet cough was assessed against bronchoscopic findings as the gold standard where BS grades ≥3 were considered abnormal (secretions present) and ≤2 considered normal (no secretions).

Age of 2 years as the cut-off was chosen because it was near the median age of the group of children examined.

**Table 5b:**

**Assessment of cough quality vs bronchoscopic findings in children aged > 2 years**

| **Assessment type**  **(clinical vs bronchoscopic findings)** | **Sensitivity** | **Specificity** | **NPV** | **PPV** | **Positive LR** |
| --- | --- | --- | --- | --- | --- |
| **Clinician**  Cough quality (wet/dry) assessed by clinician (n=62) | 1 | 0.62 | 1 | 0.68 | 2.62 |
| **Parent(s)**  Cough quality (wet/dry) assessed by parents (n=58) | 0.96 | 0.65 | 0.95 | 0.70 | 2.71 |
| **Combined** (n=61) | 1 | 0.62 | 1 | 0.68 | 2.62 |
| Cough quality (wet/dry) assessed by clinicians combined with parents. When cough was non-assessable by clinician and child has current cough, parental assessment of the cough (wet or dry) was taken. If child has no history of current cough, cough was assigned ‘dry’ | | | | | |

LR=likelihood ratio.

Specificity, sensitivity of dry and wet cough was assessed against bronchoscopic findings as the gold standard where BS grades ≥3 were considered abnormal (secretions present) and ≤2 considered normal (no secretions).
